# Epidemiology, risk factors, and clinical outcomes in severe microbial keratitis in South India

**DOI:** 10.1080/09286586.2018.1454964

**Published:** 2018-03-26

**Authors:** Jaya Devi Chidambaram, Namperumalsamy Venkatesh Prajna, Palepu Srikanthi, Shruti Lanjewar, Manisha Shah, Shanmugam Elakkiya, Prajna Lalitha, Matthew J. Burton

**Affiliations:** a International Centre for Eye Health & Clinical Research Department, London School of Hygiene and Tropical Medicine, London, UK; b Cornea Department, Aravind Eye Hospital, Madurai, Tamil Nadu, India; c Microbiology Department, Aravind Medical Research Foundation, Madurai, Tamil Nadu, India; d Cornea Department, Moorfields Eye Hospital, London, UK

**Keywords:** Acanthamoeba, aspergillus, blindness, corneal ulcer, epidemiology, fungi, fusarium, India, microbial keratitis, *Streptococcus pneumoniae*

## Abstract

***Purpose***: Here, we report risk factors associated with outcome in severe bacterial keratitis (BK), fungal keratitis (FK), and *Acanthamoeba* keratitis (AK) in India.

***Methods***: Prospective observational cohort study conducted in Aravind Eye Hospital, India. Adults presenting with severe microbial keratitis (MK) were enrolled (size ≥3 mm) and followed to 21 days post-enrolment. Ulcer clinical features were recorded at presentation. Outcomes by final visit were classified as good (completely healed or reduced infiltrate size) or poor (enlarged infiltrate size, perforated, or surgery performed).

***Results***: Of 252 participants with severe MK, 191 had FK, 18 had AK, 19 had BK, 4 had mixed BK/FK, and 20 were microbiologically negative. Median age was 50 years (interquartile range [IQR]: 37–60 years), 64% were male, 63% were agriculturalists, and 45% had no formal education. Corneal trauma occurred in 72%, and median symptom duration before presentation was 7 days (IQR: 5–15 days). Clinical features associated with FK were feathery margins (*p* < 0.001), raised profile (*p* = 0.039), or dry surface (*p* = 0.007). Hypopyon was more likely in BK (*p* = 0.001) and ring infiltrate in AK (*p* < 0.001). Ulcers with poor outcome (*n* = 106/214) were more likely to be larger (odds ratio [OR]: 1.63, 95% confidence interval [CI]: 1.30–2.05, *p* < 0.001), involve the posterior cornea at presentation (OR: 2.31, 95% CI: 1.16–4.59, *p* = 0.017), involve *Aspergillus* sp. (OR: 3.23, 95% CI: 1.26–8.25, *p* = 0.014), or occur in females (OR: 2.04, 95% CI: 1.03–4.04, *p* = 0.04). Even after treatment, 34% (*n* = 76/221) had severe visual impairment by the final visit.

***Conclusions***: Severe MK occurred predominantly in agriculturalists post-corneal trauma and often had poor outcomes. Provision of community-based eyecare may allow earlier treatment and improve outcomes.

## Introduction

Microbial keratitis (MK) can cause significant visual impairment, blindness, or even loss of the eye, thus potentially having a major impact on the individual. In India, previous studies have highlighted that MK occurs more frequently in men, those in an agricultural occupation, and is more likely following trauma to the eye.^1,2^ In particular, trauma with vegetative matter can predispose to fungal keratitis (FK), and exposure of the eyes to contaminated water is more often associated with *Acanthamoeba* keratitis (AK) in India. Although many studies in India have explored epidemiological risk factors for developing MK, very few have investigated risk factors for worse outcomes.

Lalitha et al. studied patients with FK in South India and found that primary treatment failure (i.e., progressive worsening of the ulcer despite maximal medical treatment) or corneal perforation was associated with an infiltrate size >14 mm^2^ or hypopyon at presentation or if the ulcer was culture positive for *Aspergillus* sp.^3^ Rautaraya et al. also studied bacterial keratitis (BK) in East India and also found that larger ulcer size (>25 mm^2^) or poor visual acuity at presentation was associated with poor outcome, in addition to advancing age of the patient.^4^ Titiyal et al. specifically identified risk factors for corneal perforation in a cohort of predominantly bacterial corneal ulcers in North India; these authors found that a delay in the start of the antimicrobial treatment or in fact fortified antibiotics for BK were significantly associated with perforation.^5^ One reason for delay in initiation of the correct treatment is the difficulty in making the diagnosis of BK, FK, or AK. Clinical features may play a role in differentiating these causative organisms in keratitis. Prior studies have identified feathery margins, raised surface, satellite lesions, and non-yellow infiltrate colour as more likely in FK^6,7^, ring infiltrates in AK^8^, and a well-defined border in BK (but with the exception of a wreath-like infiltrate in *Nocardia* keratitis), usually with a rapid onset of ulceration in BK.^7,8^ However, many patients present with large ulcers in India, and so the utility of these clinical signs in late-stage disease needs to be more fully evaluated.

In this study, we followed a cohort of patients presenting to Aravind Eye Hospital in South India with moderate-to-severe MK in order to understand the clinical outcomes and to assess epidemiological risk factors associated with these outcomes. We also studied the clinical features of bacterial, fungal, and *Acanthamoeba* keratitis in order to determine if any of these features were specifically associated with a particular organism.

## Materials and methods

Ethical approval was obtained for this study from the Indian Council of Medical Research, Aravind Eye Hospital Institutional Review Board, and the London School of Hygiene and Tropical Medicine Ethics Committee. The tenets of the declaration of Helsinki were adhered to in conduct of this study. Study participants gave written informed consent; illiterate participants indicated their consent with a thumbprint on the consent form, witnessed by a member of the study team (approved by above ethics committees).

Between February 2012 and February 2013, patients aged ≥18 years presenting to the Cornea Clinic at Aravind Eye Hospital (AEH), Madurai, Tamil Nadu state, South India, with MK were screened for eligibility to enter the study. Criteria for enrolment were presence of signs of MK at slit lamp examination (i.e., epithelial defect, underlying stromal infiltrate with signs of acute inflammation, e.g., conjunctival injection, anterior chamber cells, flare, or hypopyon) with stromal infiltrate ≥3 mm in longest diameter. Exclusion criteria were evidence of herpetic keratitis (based on clinical signs on slit lamp biomicroscopy or past episodes) or best-corrected Snellen visual acuity of 6/60 or worse in fellow eye. At enrolment, patients were interviewed/examined, and risk factor/clinical data were directly entered into a standard study form, which included socio-demographic details, focused clinical history, and ulcer clinical features. A standardized digital photograph was taken of the ulcer at each clinic visit. Participants were reviewed again at day 7, 14, and 21 post-enrolment (±3 days) or earlier if symptoms worsened. AK participants were followed-up in another research study.

At the baseline visit, participants underwent in vivo confocal microscopy (IVCM) using the HRT3 confocal microscope with Rostock Corneal Module (RCM, Heidelberg Engineering, Germany) as previously described.^9^ After IVCM, corneal scrapings were obtained from the base and leading edge of the ulcer for microbiological diagnosis and processed/analysed using standard methods described elsewhere.^9,10^ Culture positivity was defined as (a) growth of the same organism in at least two solid media or (b) semi-confluent growth in one solid medium at the inoculation site with the same organism identified on light microscopy.^10,11^ Organisms grown in culture were speciated using standard methods.^11–13^ BK was diagnosed only if there was a positive bacterial culture. FK was diagnosed if fungi were detected in light microscopy or IVCM and/or there was a positive fungal culture. AK was diagnosed if there was a positive culture and/or if cysts were seen within IVCM or light microscopy images.

### In vivo confocal microscopy

Prior to scraping, the corneal ulcer was imaged with the HRT3 laser scanning confocal microscope with RCM (Heidelberg Engineering, Germany) using methods previously described.^9,14^ Briefly, the cornea was anesthetized with 0.5% proparacaine (Aurocaine, Aurolab, India), and the centre and margins of the ulcer were scanned from epithelium to endothelium where possible. All of the IVCM images obtained from all of the ulcers (i.e., including culture-negative ulcers and ulcers with positive bacterial culture results) were viewed by each grader independently and assessed for the presence/absence of *Acanthamoeba* cysts or fungal filaments. For any culture and light microscopy-negative ulcers, if three or more out of five graders detected the definite presence of fungal filaments or *Acanthamoeba* cysts, these ulcers were considered diagnostically positive for FK or AK, respectively; graders were masked to the clinical and microbiological diagnosis.

### Clinical management and outcomes

All patients were treated as per standard of care at Aravind Eye Hospital by the Cornea Consultant or Cornea Fellows. Primary treatment for fungal ulcers was intensive natamycin 5% eyedrops, for BK was moxifloxacin 0.5% eyedrops, and for AK was 0.02% polyhexamethylene biguanide. Appropriate oral antifungal or antibiotic was added if the ulcer was progressing close to the limbus. Patients with moderate-to-severe MK were admitted to the hospital for in-patient treatment at the initial visit, until eyedrop frequency had reduced enough that the patient was able to instil their eyedrop therapy at home. Medical treatment was changed at the discretion of the cornea specialist if the ulcer did not respond to primary treatment. Surgical intervention (i.e., corneal glue, intrastromal antifungal injection, and therapeutic penetrating keratoplasty as required) was offered in the event of corneal perforation or clinical worsening despite maximal medical treatment. Clinical outcomes were defined as: “healed” if by the final visit there was <0.5 mm epithelial defect and evidence of scar tissue either wholly or partly replacing the stromal infiltrate as observed with slit lamp biomicroscopy; “improving” if the stromal infiltrate diameter was less than 20% of the original diameter on slit lamp examination; “worse” if by the final visit the ulcer was the same size or larger than the original diameter; “perforated” if there was evidence of full-thickness corneal perforation; or flat anterior chamber ± evidence of suspected corneal perforation (i.e., iris plugging a possible corneal perforation site or seidel-positive fluorescein dye test showing a leak of aqueous humour through suspected perforation). “Good” outcome was defined as a composite of those who had “healed” and “improved” outcomes. “Poor” outcome was a composite of those who had “worse” and “perforated” outcomes.

### Statistical analysis

Socio-demographic and clinical feature data were compared between organism groups and statistical significance assessed using the chi-square test for proportions and Kruskal–Wallis test for non-parametrically distributed variables (age, symptom duration, and ulcer size). Ulcer size was defined as the geometric mean of the longest diameter of the stromal infiltrate and its perpendicular diameter. Causative organism prevalence for severe MK over the study period was calculated using the denominator as the total number of eligible study participants with severe MK and not the total number of patients with MK of any size seen at AEH over the study period. Multivariable logistic regression analysis was performed to assess clinical features associated with FK, *Aspergillus* versus *Fusarium* keratitis, as well as risk factors associated with worse versus good outcome (adjusted for age and gender). Forwards stepwise regression analysis was used, and variables assessed for significance against the full model using likelihood ratio testing. A significance level of *p* < 0.1 was used for initial inclusion in the model and for *p* ≤ 0.05 in likelihood ratio testing (against the full model) for retention in the model. All analyses were performed in Stata v12.1 (StataCorp, Texas, USA).

## Results

Among the 252 eligible participants enrolled in the study, the organisms detected were fungi in 191 (77%), *Acanthamoeba* sp. in 18 (7%), bacteria in 19 (7%), microbiologically negative (i.e., no organism detected by culture, light microscopy, or IVCM) in 20 (7%), and mixed bacterial/fungal infection in 4 (2%). In the pure fungal cultures, the most frequently detected fungi were *Fusarium* sp. (*n* = 75/191; 39%) and *Aspergillus* sp. (*n* = 35/191; 18%). In pure bacterial ulcers, the main bacteria identified were *Streptococcus pneumoniae* (*n* = 9/19; 47%), *Pseudomonas aeruginosa* (*n* = 4/19; 21%), and *Nocardia* sp. (*n* = 3/19; 16%).

### Socio-demographic risk factors for bacterial, fungal, and *Acanthamoeba* keratitis

The median age of all participants was 50 years (interquartile range [IQR] 37–60 years), of whom 64% were male (*n* = 162). Most patients were agricultural workers (63%, *n* = 159/251), and 45% of participants had not undergone any formal education (*n* = 111/246). There were no significant differences in age, gender, agricultural occupation, or educational status between the FK, BK, AK, and microbiologically negative groups (Table 1). A greater proportion of the microbiologically negative group were current tobacco users compared to BK, FK, and AK (33%, *n* = 6/18; *p* = 0.047 – see Table 1). Corneal trauma was sustained in 72% of participants immediately prior to onset of the ulcer (*n* = 181/252). The source of trauma was vegetative matter in 46% (*n* = 82/180); vegetative trauma occurred more frequently in the BK group (76% of BK patients with corneal trauma, *n* = 10/13), rather than FK, AK, or microbiologically negative ulcers (*p* = 0.033, Table1) and was mainly in ulcers caused by *S. pneumoniae* (*n* = 5/10) or *Nocardia* sp. (*n* = 2/10).

**Table 1.** Socio-demographic and clinical risk factors associated with bacterial, fungal, and *Acanthamoeba* keratitis at presentation.

Risk factor	All microbial keratitis	Bacterial keratitis	Fungal keratitis	*Acanthamoeba* keratitis	No organism	*p*-Value*
	*N* = 252	*N* = 19	*N* = 191	*N* = 18	*N* = 20	
**Socio-demographic**						
Age, years (median, IQR)	50 (37–60)	60 (35–65)	50 (36–58)	39 (34–55)	53 (43–61)	0.066
Gender (% male)	162 (64)	12 (63)	123 (64)	11 (61)	13 (65)	0.993
Occupation: no. agricultural workers (%)	159/251 (63)	15 (79)	121 (64)	11 (61)	10 (50)	0.311
Had primary education or greater (%)	135/246 (55)	8 (42)	107/186 (57)	9/17 (53)	10 (50)	0.577
Current tobacco use (%)	34/234 (14)	4/16 (25)	23/178 (13)	1 (6)	6/18 (33)	0.047
**History**						
Symptom duration before presentation to AEH, days (median, range)	7 (5–15)	9 (5–15)	7 (4–11)	30 (20–60)	10 (7–21)	<0.001
Symptom duration before presentation to any healthcare provider (HCP), days (median, range)	2 (0–3)	2 (0.5–5)	2 (0–3)	2 (0–7)	0 (0–3)	0.547
History of eye trauma (%)	181 (72)	13 (68)	134 (70)	13 (72)	17 (85)	0.563
Trauma with vegetative matter (%)	82/251 (33)	10/13 (77)	56/133 (42)	3/13 (23)	9/17 (53)	0.033
Prior antifungal use (%)	125/220 (57)	7/16 (44)	98/169 (58)	10 (59)	8/15 (53)	0.727
Prior antibiotic use	162/220 (74%)	11/16 (69%)	123/169 (73%)	14/17 (82%)	11/15 (71%)	0.825
Prior steroid use (%)	25/220 (11)	1/16 (6)	16/169 (9)	7/17 (41)	1/15 (7)	0.001
Traditional medicine use (%)	48 (19)	4 (21)	37 (19)	5 (28)	2 (10)	0.578
Plant-based traditional medicine used (%)	21/47 (45)	2/4 (50)	15/36 (42)	3/5 (60)	1/2 (50)	0.879
Prior MK in affected eye (%)	9 (4)	1 (5)	8 (4)	0 (0)	0 (0)	0.627
Diabetes mellitus (%)	17 (7)	0 (0)	15 (8)	0 (0)	1 (5)	0.356
**Initial clinical features**						
Stromal infiltrate size, mm^2^ (median, IQR)	4.5 (3.5–5.9)	3.9 (3.2–6.1)	4.5 (3.3–5.5)	6.8 (5.3–8.0)	5.2 (4.0–6.1)	<0.001
Posterior 1/3 of cornea affected at presentation (%)	168/251 (67)	14 (74)	123 (64)	12 (71)	17 (85)	0.261
Entropion, Ectropion, or lagophthalmos present (%)	12 (5)	2 (10)	8 (4)	0 (0)	2 (10)	0.310
Blocked tear duct (%)	19 (7)	4 (21)	12 (6)	0 (0)	3 (15)	0.039

*Bacterial, fungal, and *Acanthamoeba* groups compared for statistically significant differences using chi-square test (for proportions) or Kruskal–Wallis test (non-parametrically distributed variables).

In all ulcers, the median symptom duration prior to presentation was 7 days (IQR: 5–15 days), but this was significantly longer in AK compared to all other groups (30 days, IQR: 20–60 days, *p* < 0.001; Table 1), as we have previously reported.^9^ The majority of patients (90%, *n* = 226/252) had sought help from another healthcare provider (HCP) prior to presentation at AEH, including other ophthalmologists (65%, *n* = 163/252), general physicians (8%, *n* = 21/252), pharmacists (7%, *n* = 17/252), and traditional medicine healers (19%, *n* = 48/252); 25 patients (10%) saw more than one HCP. At presentation, 220 patients were using antimicrobial treatment: 71 were using topical antibiotics, 34 topical antifungals alone, and 91 were using both antifungal and antibiotic. Topical steroids were being used by 25 patients at presentation, and this was particularly the case in AK (*n* = 7/18; 39%, *p* < 0.001; Table 1). Traditional medicines were used as initial treatment for the ulcer in 19% of participants (*n* = 48/252); the most frequent remedies used were topically applied breast milk (*n* = 24) or plant-based oils/flower extracts (*n* = 22, e.g., castor oil, *n* = 8). Use of plant-based traditional medicines was not significantly associated with FK or any other causative organism (*p* = 0.879, Table 1).

Patients reported their main reasons for delay in seeing an ophthalmologist as a lack of pain or reduced vision causing the patient to believe the eye problem was not serious (51%, *n* = 69/134), lack of availability of a person to escort the patient to the eye hospital (32%, *n* = 43/134), lack of finances for travel costs (6%, *n* = 8/134), other family, work, or travel commitments (7%, *n* = 9/134), and lack of knowledge of local eyecare services (4%, *n* = 5/134).

### Ocular and systemic risk factors

With regard to systemic medical conditions, diabetes mellitus was present in 17 patients (Table 1), 15 of whom had FK. Presence of a blocked tear duct occurred more frequently in BK than in FK, AK, or microbiologically negative ulcers as shown in Table 1 (21% in BK, *n* = 4/19, *p* = 0.039). All of these bacterial ulcers were culture positive for *S. pneumoniae*.

Overall, presenting best-corrected visual acuity in the affected eye fulfilled the WHO criteria for moderate or severe visual impairment in 71.3% of patients (*n* = 174/244; detailed in Table 2). In the unaffected eye at presentation, 10.7% of patients had moderate visual impairment (*n* = 26/244). Stromal infiltrate size was also large at presentation for all MK patients (median 4.5 mm^2^, IQR: 3.5–5.9 mm^2^) and with deep involvement of the posterior third of the cornea in 67% of ulcers (*n* = 168/251). Ulcer size was significantly greater in AK than in BK, FK, or microbiologically negative ulcers (median 6.8 mm^2^ stromal infiltrate diameter, IQR: 5.3–8.0 mm^2^, *p* < 0.001; Table 1).

**Table 2.** Proportion of study participants with mild, moderate, or severe visual impairment (VI) or blindness in affected eye at presentation and at final visit, as per the World Health Organization criteria.

Risk factor	Totals (%)	Bacterial keratitis (BK) (%)	Fungal keratitis (FK) (%)	*Acanthamoeba* keratitis (AK) (%)	No organism (%)	*p*-Value*
**Visual acuity (Snellen VA) at presentation**	***N* = 244**	***N* = 19**	***N* = 189**	***N* = 16**	***N* = 20**	
Normal (<6/9)	22 (9)	1 (5)	19 (10)	1 (6)	1 (5)	0.773
Mild VI (6/9 to 6/18)	24 (10)	1 (5)	21 (11)	0 (0)	2 (10)	0.468
Moderate VI (>6/18 to 6/60)	35 (14)	2 (10)	31 (16)	1 (6)	1 (5)	0.369
Severe VI (>6/60 to 3/60)	139 (57)	13 (68)	102 (54)	11 (69)	13 (65)	0.370
Blind (>3/60)	24 (10)	2 (10)	16 (8)	3 (19)	3 (15)	0.485
**VA at final visit**	***N* = 221**	***N* = 18**	***N* = 185**	-	***N* = 18**	
Normal	32 (14)	0 (0)	30 (16)		2 (11)	0.155
Mild VI	43 (19)	3 (17)	36 (19)	-	4 (22)	0.915
Moderate VI	34 (15)	2 (11)	28 (15)	-	4 (22)	0.641
Severe VI	76 (34)	7 (39)	65 (35)	-	4 (22)	0.489
Blind	36 (16)	6 (33)	26 (14)	-	4 (22)	0.413

*Proportion of BK, FK, and AK groups within each visual acuity category compared with all others for statistically significant differences using chi-square test.

### Ulcer appearance at presentation and causative organism

At the first visit, fungal ulcers were more likely to have feathery margins (*p* < 0.001), raised profile (*p* = 0.039), and dry texture of surface slough (*p* = 0.007) compared to the BK and AK groups in univariable analysis (Table 3). Logistic regression analysis of fungal ulcers versus all others showed that feathery margins were strongly associated with fungal ulcers (odds ratio [OR]: 4.47, 95% confidence interval [CI]: 2.10–9.50, *p* < 0.001) and ring infiltrate associated with non-fungal ulcers (OR: 0.43, 95% CI: 0.20–0.92, *p* = 0.029). A higher proportion of AK patients had a ring infiltrate (89%, *n* = 16/18), than in FK or BK (*p* < 0.001). Bacterial ulcers were more likely to have a hypopyon (*p* = 0.002; Table 3), and in fact all ulcers caused by *S. pneumoniae* in this study had a hypopyon at presentation (*n* = 10 [9 pure BK and 1 mixed BK/FK]).

**Table 3.** Clinical features in bacterial, fungal, and *Acanthamoeba* keratitis at presentation.

Ulcer appearance	Bacterial keratitis, BK (*n* = 19) (%)	Fungal keratitis, FK (*n* = 191) (%)	*Acanthamoeba* keratitis, AK (*n* = 18) (%)	*p*-Value*
Feathery margin	5 (26)	134 (70)	2 (11)	<0.001
Raised profile	3 (16)	48 (25)	0 (0)	0.039
Dry texture of slough	1 (5)	77 (40)	5 (28)	0.007
Satellite lesions	5 (26)	70 (37)	2 (11)	0.070
Non-yellow infiltrate	12 (63)	141 (74)	16 (89)	0.197
Pigmented ulcer	0 (0)	9 (5)	0 (0)	0.404
Ring infiltrate	8 (42)	54 (28)	16 (89)	<0.001
Perineural infiltrate	0 (0)	1 (0.5)	1 (6)	0.083
Corneal neovascularization	5 (26)	22 (11)	3 (17)	0.172
DM folds	6 (32)	94 (49)	6 (33)	0.172
Endothelial plaque	10 (53)	75 (39)	4 (22)	0.164
Hypopyon	17 (89)	120 (63)	6 (33)	0.002
Cells and/or flare in AC	11/12 (92)	102/148 (69)	6/12 (50)	0.085
Keratic precipitates	6 (32)	55/183 (30)	4/16 (25)	0.900

*Comparison of all three groups (BK, FK, and AK) to assess for statistically significant differences using chi square test.

Within FK, comparing ulcers caused by *Fusarium* sp. with *Aspergillus* sp., we found that *Fusarium* ulcers were more likely to have feathery margins (OR: 4.55, 95% CI: 1.92–10.75, *p* = 0.001) or a non-yellow infiltrate (OR: 4.42, 95% CI: 1.78–10.95, *p* = 0.001) in the univariable logistic regression analysis. *Aspergillus* ulcers were more likely to have a raised surface (OR: 2.67, 95% CI: 1.10–6.44, *p* = 0.029) or a hypopyon (OR: 2.98, 95% CI: 1.16–7.67, *p* = 0.024) compared to *Fusarium* ulcers in univariable analysis. There was no significant difference between *Fusarium* or *Aspergillus* ulcers for the presence of satellite lesions, ring infiltrate, Descemet’s membrane folds, or an endothelial plaque. Multivariable analysis found that only feathery margins (OR: 3.56, 95% CI: 1.45–8.76, *p* = 0.006) and non-yellow infiltrate colour (OR: 3.28, 95% CI: 1.26–8.56, *p* = 0.015) remained significantly associated with *Fusarium* infection.

### Risk factors associated with clinical outcome

Clinical outcome data were obtained for 227 participants; no outcome data were available for seven participants who were lost to follow-up after the first visit (*n* = 5 FK, *n* = 1 BK, and *n* = 1 microbiologically negative) or for the 18 participants with AK (who were followed in another study). By the final visit, 110 participants had a good outcome (40 healed and 70 were improving) and 117 had a poor outcome (i.e., perforated/required corneal glue, *n* = 42, or worsened, *n* = 75). Overall, by the final visit, best-corrected visual acuity measurements found that most MK patients had severe visual impairment (severe VI, 34%, *n* = 76/221), and 16% (*n* = 36/221) fulfilled the WHO criteria for blindness (Table 2).

Participants with a poor outcome presented with a significantly longer symptom duration (median 9 days vs. 7 days in good outcome group, *p* < 0.001; Table 4), worse visual acuity (median logMAR 1.8 vs. 1.7 in good outcome, *p* < 0.001; Table 4), and larger ulcers (median infiltrate size 5.2 mm^2^ vs. 3.8 mm^2^, *p* < 0.001; Table 4). A greater proportion of those with a poor outcome had involvement of the posterior cornea at presentation (80% vs. 53%, *p* < 0.001; Table 4) or presence of a hypopyon (74% vs. 55% of those with a good outcome, *p* = 0.004). Multivariate analysis revealed that the main epidemiological risk factors most significantly associated with poor outcome were female gender (OR: 2.04, 95% CI: 1.03–4.04, *p* = 0.04; Table 5), no formal education (OR: 2.30, 95% CI: 1.14–4.62, *p* = 0.019; Table 5), and symptom duration (OR: 1.05, 95% CI: 1.00–1.10, *p* = 0.032; Table 5). Ulcer features also associated with poor outcome were larger ulcer size at presentation (OR: 1.63, 95% CI: 1.30–2.05, *p* < 0.001), as well as involvement of the posterior cornea (OR: 2.31, 95% CI: 1.16–4.59, *p* = 0.017) and culture-positive result for *Aspergillus* sp. (OR: 3.23, 95% CI: 1.26–8.25, *p* = 0.014; Table 5). Several socio-demographic features were significantly associated with good outcome, including younger age at presentation (median age 45 years vs. 53 years in poor outcome group, *p* = 0.036; Table 4), male gender (71% vs. 58% with poor outcome, *p* = 0.031), and educational status of primary school level or beyond (65% vs. 46% in poor outcome, *p* = 0.004).

**Table 4.** Risk factors associated with clinical outcomes in severe microbial keratitis (*groups compared with chi-square test for proportions and Kruskal–Wallis for continuous non-parametrically distributed variables).

Outcome risk factor	Worse (*n* = 117)	Good (*n* = 110)	*p*-Value*
**Socio-demographic**			
Age, years (median, IQR)	53 (40–60)	45 (35–57)	0.036
Gender (no. male, %)	68 (58)	79 (71)	0.031
Occupation: agricultural (%)	82 (70)	62/109 (57)	0.039
Primary education or more (%)	53 (46)	69 (65)	0.004
Current smoker/tobacco use (%)	14 (13)	18 (17)	0.392
**History**			
Symptom duration, days (median, range)	9 (5–15)	7 (4–10)	<0.001
Any eye trauma (%)	84 (72)	80 (73)	0.875
Trauma with vegetative matter (%)	44 (38)	32/109 (29)	0.190
Prior antifungal use (%)	61/102 (60)	50/94 (53)	0.351
Prior antibiotic use (%)	77 (75)	64 (68)	0.249
Prior steroid use (%)	9 (9)	8 (8)	0.938
Traditional medicine use (%)	20 (17)	22 (20)	0.573
Plant based (%)	7/19 (37)	10/22 (45)	0.577
Seen by another healthcare provider first (%)	103 (88)	98 (89)	0.803
Prior microbial keratitis (%)	6 (5)	3 (3)	0.354
Diabetes mellitus (%)	12 (10)	4 (4)	0.051
**Clinical features at presentation**			
Best-corrected visual acuity-affected eye (median logMAR, IQR)	1.8 (1.7–1.8)	1.7 (0.46–1.8)	<0.001
Stromal infiltrate size, mm^2^ (median, IQR)	5.2 (4.0–6.5)	3.8 (3.1–4.7)	<0.001
Posterior 1/3 of cornea involved (*N*, %)	94 (80)	58 (53)	<0.001
Presence of hypopyon (%)	86 (74)	61 (55)	0.004
**Organism**			
Bacteria (excluding mixed BK/FK) (%)	10 (9)	8 (7)	0.695
*Streptococcus pneumoniae* (%)	7 (7)	2 (2)	0.108
Fungi (excluding mixed BK/FK) (%)	95 (86)	91 (84)	0.661
*Fusarium* spp. (%)	28 (24)	46 (42)	0.004
*Aspergillus* spp. (%)	22 (19)	8 (7)	0.010

**Table 5.** Univariate and multivariate logistic regression analyses of risk factors associated with worse outcome in severe microbial keratitis (*adjusted for age and gender).

Risk factor	Univariable OR* (95% CI)	*p*-Value	Multivariable OR (95% CI)	*p*-Value
Age	1.09 (1.00–1.04)	0.048	0.99 (0.97–1.01)	0.539
Female gender	1.84 (1.05–3.20)	0.032	2.04 (1.03–4.04)	0.040
Presenting ulcer infiltrate size (mm^2^)	1.80 (1.45–2.23)	<0.001	1.63 (1.30–2.05)	<0.001
*Aspergillus* sp. isolated	2.87 (1.19–6.88)	0.018	3.23 (1.26–8.25)	0.014
Posterior corneal involvement	3.33 (1.82–6.11)	<0.001	2.31 (1.16–4.59)	0.017
No formal education	1.68 (0.92–3.05)	0.089	2.30 (1.14–4.62)	0.019
Symptom duration (days)	1.07 (1.03–1.12)	0.001	1.05 (1.00–1.10)	0.032
Vegetative trauma	1.68 (0.94–3.02)	0.079	-	-

Analysis of the outcomes for the most frequent causative organisms showed that 62% of *Fusarium* sp. ulcers had a good outcome (*n* = 46/74), versus only 23% in *Aspergillus* sp. ulcers (*n* = 8/35), and 22% in *S. pneumoniae* ulcers (*n* = 2/9, i.e., *n* = 2/8 pure *S. pneumoniae* keratitis and *n* = 0/1 mixed infection with fungus; Table 6). With regard to perforation, 18% of *Fusarium* ulcers perforated (*n* = 13/74), versus 20% of *Aspergillus* ulcers (*n* = 7/35), and 56% of *S. pneumoniae* ulcers perforated or required corneal glue (*n* = 5/9, i.e., *n* = 4/8 pure *S. pneumoniae* keratitis and *n* = 1/1 mixed infection with fungus; Table 6). Overall, patients who developed corneal perforation had significantly longer symptom duration prior to presentation (median 10 days, IQR: 6–15, *p* = 0.008) compared to all other ulcers (median 7 days, IQR: 4–10). The ulcer itself was significantly larger at presentation in the perforated group (5.3 mm^2^ in perforated ulcers, IQR: 3.9–6.9 mm^2^ vs. 4.2 mm^2^ all others, IQR: 3.3–5.3 mm^2^, *p* = 0.001) and a greater proportion of perforated ulcers already involved the posterior third of the cornea at the first visit (90% in perforated ulcers vs. 62% all others, *p* < 0.001). Initial visual acuity was also significantly poorer in the perforation group (median logMAR 1.8, IQR: 1.7–1.8, *p* = 0.001).

**Table 6.** Organisms identified by culture, light microscopy, and *in vivo* confocal microscopy by clinical outcome.

Organisms	Perforated or glued (*n* = 42)	Worse (*n* = 75)	Improving (*n* = 70)	Healed (*n* = 40)
**Bacteria (*n* = 18)**	***n* = 6**	***n* = 4**	***n* = 4**	***n* = 4**
*Streptococcus pneumoniae*	4	2	1	1
*Streptococcus viridans*	1	0	0	0
*Staphylococcus epidermidis*	0	1	0	0
*Nocardia* sp.	0	0	2	1
*Pseudomonas aeruginosa*	1	1	0	2
*Aeromonas* sp.	0	0	1	0
**Fungi (*n* = 186)**	***n* = 32**	***n* = 63**	***n* = 59**	***n* = 32**
*Aspergillus* sp.	7	20	7	1
*Fusarium* sp.	13	15	25	21
*Alternaria* sp.	0	0	1	0
*Bipolaris* sp.	0	0	1	0
*Curvularia* sp.	0	1	2	1
*Cylindrocarpon* sp.	1	1	0	0
*Exserohilum* sp.	2	2	0	1
*Lasiodiplodia* sp.	0	1	1	0
Unidentified hyaline fungi	1	10	4	0
Unidentified dematiaceous fungi	2	3	5	1
Light microscopy positive for fungi, culture negative	5	7	10	5
Light microscopy and culture negative for fungi, but IVCM positive for fungi	1	3	3	2
Mixed bacterial/fungal* (*n* = 4)	***n* = 1**	***n* = 2**	***n* = 1**	***n* = 0**
*Streptococcus pneumoniae*	1	0	0	0
*Streptococcus viridans*	0	1	1	0
*Staphylococcus epidermidis*	0	1	0	0
**Micro/IVCM negative (*n* = 19)**	***n* = 3**	***n* = 6**	***n* = 6**	***n* = 4**
Light microscopy, culture, and IVCM negative for any organism	3	6	6	4

*Mixed infections were culture positive for bacteria (species shown in table) and also positive for fungi detected only in light microscopy and/or *in vivo* confocal microscopy.

## Discussion

In this prospective study, we have explored epidemiological risk factors and clinical features associated with severe BK, FK, and AK and described MK clinical outcomes. In severe MK, the outcomes for many patients can be poor, with corneal perforation in up to 30% and loss of the eye in up to 25%.^15,16^ Numerous prior publications have examined the epidemiology of MK throughout India. Our results also show that agricultural workers are predominantly affected and that exposure to corneal trauma, frequently with vegetative matter, is an important risk factor for onset of MK. These individuals were often male and within the working age-group in this study. Most previous reports have noted an association between vegetative trauma and eyes with fungal infection; however, our findings indicate an association with BK.^2,10^ This highlights the fact that corneal injury with vegetative matter may provide a route of entry for other pathogens such as *S. pneumoniae* that are not classically known as plant pathogens. Although most participants in this study presented to the eye hospital approximately 7 days or more after onset of symptoms, many had visited an HCP much earlier, sometimes within the first day of developing symptoms. However, many patients were not placed on appropriate antimicrobial treatment following this initial assessment; particularly in the case of AK patients, many were started on topical steroids without anti-acanthamoeba treatment prior to presentation at the eye hospital, which has been associated with worse visual outcome in AK.^17^

We found in our study population that poor outcomes occurred predominantly in those of an older age, female gender, and with no formal education. Local eyecare service providers could aim to reduce these health inequalities in the future when planning services. In addition, larger ulcers with longer symptom duration had a poor outcome, as reported by others^3,4^, highlighting the need for patient education to encourage individuals to seek eyecare early on in the course of disease to improve final visual outcomes. Many patients reported that they either did not take their initial ocular symptoms seriously or that they had difficulties in organizing travel to the eye hospital along with a person to accompany them. Other studies have analysed barriers in the uptake of eyecare services in rural India and found similar reasons to those reported in our study.^18,19^ Provision of antimicrobial eyedrop therapy such as natamycin in community-based clinics may alleviate these issues and allow correct antimicrobial treatment to be instituted under ophthalmic supervision early on in the course of disease.

We have found specific epidemiological risk factors (female gender or no formal education) and ulcer-related risk factors (culture positive for *Aspergillus* sp., involvement of the posterior cornea, and larger ulcer size at presentation) in the multivariate analysis that were associated with poor outcome in our South Indian, mainly rural, study population with moderate-to-large MK. The greatest risk factor for poor outcome in our study was *Aspergillus* keratitis. Presence of one or more of these specific risk factors at presentation may be of prognostic value. Prognostic risk scoring models have been developed and used successfully for ocular trauma as well as other diseases, e.g., myocardial infarction or stroke, and can help the clinician to identify more highly at-risk patients at presentation.^20,21^ Future studies with larger samples size could be used to formally assess the risk factors that we have described in the form of a prognostic risk score for MK.

The etiological agent in larger ulcers can be difficult to diagnose based on clinical features alone.^7^ We found that even in moderate-to-large ulcers, the presence of feathery margins, raised profile, and dry surface are more likely to occur in FK, as also shown by others.^6,7^ Unlike previous studies that have assessed features specific to *Fusarium* sp. or *Aspergillus* sp., we found that feathery margins were more associated with *Fusarium* sp. and raised profile or hypopyon with *Aspergillus* sp.^22^ Most of the bacterial ulcers in this study were caused by *S. pneumoniae*, and not only presented with hypopyon, but many ultimately perforated.

The main limitation of this study was that we only included moderate-to-severe ulcers, which limit the wider generalizability of the study findings. However, these ulcers are frequently the most difficult to manage, with the worse outcomes; therefore, to identify the organisms involved and risk factors associated with such poor outcomes is an important step towards developing appropriate solutions for MK. Also, data were collected in a single institution that is a tertiary referral centre based in Madurai, South India. Although there is increasing urbanization of the villages/towns immediately surrounding Madurai, most of the villages in this region have remained rural, as indicated by census of India data.^23^ Therefore, more severe cases may have presented to the Cornea Clinic at this institution, and the catchment area for the institution may include a greater rural population than other institutions.

In summary, in this study, we have described several risk factors found in South Indian patients with moderate-to-severe MK, which include female gender, no formal education, and increased severity of the ulcer at presentation all associated with worse outcomes. Late-stage MK resulted in severe visual impairment or blindness in half of the study participants by the final visit. Community-based prevention strategies aimed at women and those with low educational status might therefore improve visual outcomes by providing prophylactic antimicrobial treatment for corneal abrasions prior to the onset of MK.
